# Oral Acarbose Inhibits Baseline Salivary Amylase and Blunts Early Postprandial Glycemia: Randomized Cross-Over Study

**DOI:** 10.2196/98441

**Published:** 2026-11-06

**Authors:** Airi Sekine, Kei Nakajima

**Affiliations:** 1 Department of Nutritional Science, Faculty of Food and Nutritional Sciences, Japan Women's University, 2-8-1 Mejirodai, Bunkyo-ku, Tokyo, 112-8681, Japan, 81 0359813427 ext 3427, 81 5981-3427; 2 Saitama Medical Center, Department of Endocrinology and Diabetes, Saitama Medical University, Kawagoe, 350-8550, Japan

**Keywords:** acarbose, salivary amylase, SAA, starch loading, postprandial blood glucose, salivary α-amylase

## Abstract

This study investigated whether salivary α-amylase (SAA) inhibition by acarbose affects postprandial blood glucose (BG) in healthy individuals. This single-blinded, placebo-controlled, randomized cross-over trial included 4 conditions: trial 1 (T1), placebo (oligosaccharide); trial 2 (T2), swallowing 50 mg of acarbose; and trials 3 and 4 (T3 and T4), simultaneous intake of starch (150 g of rice) with 50 mg or 100 mg of acarbose, respectively. Fifteen young, healthy women were enrolled. Peak ΔBG occurred at 30 min in both T1 and T2 and at 60 min in T3. ΔBG at 30 min was significantly lower in T3 and T4 than in T2 (both *P*<.05) without trial order effect, while BG areas under the curve did not differ between T2 and T3. Inhibition of SAA by oral acarbose reduced the early postprandial increase in BG compared with intact SAA activity in healthy individuals.

## Introduction

α-Glucosidase inhibitors reduce postprandial glucose excursions by inhibiting α-glucosidase enzymes in the small intestine [1]. Among these agents, only acarbose—a complex oligosaccharide—inhibits α-amylase [1,2], which is produced in both the pancreas and salivary glands in humans [3]. However, the effect of acarbose inhibition of oral salivary α-amylase (SAA) during starch chewing (an unusual mode of drug intake) on postprandial blood glucose (BG) has not been investigated. Therefore, this pilot study examined the effect of oral acarbose action on post–starch-load BG and SAA in young, healthy women.

## Methods

### Study Design

This was a single-blinded, placebo-controlled, randomized cross-over study comprising 4 trials.

### Ethical Considerations

The study was conducted in accordance with the Declaration of Helsinki and was approved by the Institutional Review Board of the Ethics Committee of Japan Women’s University (ID number RR703). Written informed consent was obtained from each participant.

### Participants

Fifteen healthy Japanese female university students aged 21 to 22 years were recruited. All participants were nonsmokers and non–heavy drinkers. Individuals with metabolic disorders, including diabetes, dyslipidemia, and chronic constipation, were excluded. The 4 trials were conducted in a randomized, participant-blinded, and counterbalanced manner with a washout period of 1 week or more, preventing potential confounding from period effects.

In pretests, SAA was substantially inhibited by 50 mg of oral acarbose in all participants (Multimedia Appendix 1).

Trial 1 (T1) comprised placebo ingestion (50 mg oligosaccharide). Rice was ingested immediately after chewing encapsulated lactosucrose (ENSUIKO Sugar Refining Co Ltd), a nondigestible oligosaccharide, for 5 to 10 seconds.

Trial 2 (T2) involved swallowing encapsulated acarbose (50 mg) with a small amount of water (SAA intact). Encapsulated acarbose was swallowed without chewing, passing through the oral cavity in the usual manner, that is, rice was then immediately ingested, chewed, and swallowed naturally within 4 minutes.

Trial 3 (T3) comprised simultaneous ingestion of encapsulated acarbose (50 mg) and rice (SAA inhibited). Encapsulated acarbose was chewed for 10 seconds and swallowed naturally.

Trial 4 (T4) comprised simultaneous ingestion of encapsulated acarbose (100 mg) and rice (SAA inhibited). The procedure was identical to T3.

In the morning after overnight fasting, each test was conducted following 5 minutes of seated rest.

For starch loading, participants ingested 150 g of commercially available heated retort white rice (energy: 209 kcal, carbohydrate: 48.8 g, protein: 2.9 g, fat: 0 g) prepared from a precooked rice pack. Saliva samples were collected every 30 min up to 120 min using oral swabs. SAA, which was adjusted for salivary protein concentration, and BG were measured as previously described [4].

Sample size was based on a moderate-to-large within-participant effect size (*d*_*z*_ ≥0.7), 80% power, and *α*=.05. Changes in SAA and BG were calculated as Δ values, and areas under the curve (AUCs) were calculated using the trapezoidal rule [5]. A mixed-effects model was used to assess the trials and the order (carryover) effects. Statistical analyses were performed using SAS Enterprise Guide in the SAS system, version 9.4 (SAS Institute). A *P* value <.05 was considered significant.

## Results

The participants’ mean BMI was 20.2 (SD 1.4) kg/m^2^. No adverse events occurred throughout the trials. The peak ΔBG occurred at 30 min in T1 and T2, occurred at 60 min in T3, and was not observed in T4 (Figure 1). ΔBGs at all time points and BG AUCs in T1 were significantly higher than those in the other trials. Mean ΔBG at 30 min was significantly lower in T3 (6.9, SE 2.3 mg/dL) and T4 (0, SE 2.0 mg/dL) than in T2 (17.9, SE 5.1 mg/dL) (both *P*<.05, post hoc test; overall ANOVA *P*<.0001) without significant carryover effects of trial order (*P*=.94). ΔBG at 60 min was significantly higher in T3 than in T4 (order effect; *P*=.49). However, no significant difference in BG AUCs was observed between T2 and T3 (Table 1).

**Figure 1.**
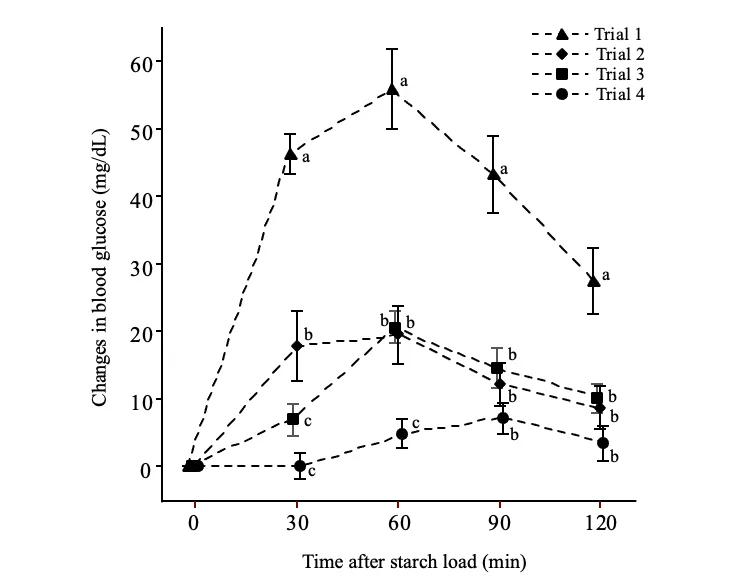
Changes in blood glucose after starch loading. Symbols and bars indicate means and SEs. The earliest peak from baseline occurred at 30 minutes in T1 and T2, occurred at 60 minutes in T3, and was not observed in T4. Means sharing a common letter are not significantly different (*P*≥.05) at a given time point. Changes in BG were significantly higher in T1 at all time points compared with the other trials (all *P*<.05, post hoc test, Student-Newman-Keuls test). The linear mixed-effects model revealed a significant trial effect on ΔBG at 30 and 60 minutes (both *P*<.0001), with no significant carryover effect of trial order (*P*=.94 and *P*=.49, respectively).

Positive linear trends were observed between ΔSAA and increasing time points in T1, T2, and T3 (*P*=.01‐.04) but not in T4 (Multimedia Appendix 2). No significant differences in ΔSAA at any time point or in SAA AUCs were observed among the 4 trials (post hoc test) (Table 1).

The incremental AUC (iAUC) was defined as the area above the fasting BG level, with negative values set to 0. The net AUC (nAUC), which includes both positive and negative areas relative to baseline, was calculated by subtracting the area below baseline from the total AUC.

**Table 1.** Blood glucose (BG) areas under the curve (AUCs) and salivary α-amylase (SAA) AUCs in 4 trials.

Measurement	Mean (SD)	Median (IQR)	*P* value for ANOVA	*P* value for differences between trials (post hoc test)^a^	*P* value for trial’s order effect^b^	*P* value for trial’s effect^b^
BG iAUCs^c^ (mg · h/dL)			<.0001		.62	<.0001
T1	79.6 (26.2)	71.5 (56.8 to 91.3)		a		
T2	26.9 (17.3)	26.8 (18.0 to 40.5)		b		
T3	23.4 (11.6)	21.3 (13.0 to 35.8)		b		
T4	8.3 (7.1)	6.9 (2.7 to 13.2)		c		
BG nAUCs^d^ (mg · h/dL)			<.0001		.58	<.0001
T1	79.6 (26.2)	71.5 (56.8 to 91.3)		a		
T2	27.4 (20.7)	28.3 (15.8 to 46.0)		b		
T3	23.6 (11.7)	20.8 (12.8 to 35.3)		b		
T4	5.5 (9.0)	5.5 (−0.3 to 10.8)		c		
SAA iAUCs^c^ (U · h/L)			.42		.63	.58
T1	39.3 (23.9)	38.0 (20.2 to 60.6)		d		
T2	38.3 (31.8)	28.8 (11.7 to 69.7)		d		
T3	31.5 (18.9	26.4 (17.0 to 51.0)		d		
T4	26.5 (18.9)	25.8 (12.0 to 36.5)		d		
SAA nAUCs^d^ (U · h/L)			.46		.65	.73
T1	37.9 (26.0)	38.0 (20.2 to 60.6)		d		
T2	34.6 (36.2)	28.8 (9.4 to 69.7)		d		
T3	31.3 (19.2)	26.4 (17.0 to 51.1)		d		
T4	22.8 (23.3)	25.3 (12.0 to 36.5)		d		

a Letter “a” indicates that T1 was significantly higher than all other trials (*P*<.05, Student-Newman-Keuls test). Letter “b” indicates that T2 and T3 showed no significant difference between each other (*P*≥.05) but were significantly lower than T1 and significantly higher than T4 (*P*<.05). Letter “c” indicates that T4 was significantly lower than all other trials (*P*<.05). Letter “d” indicates statistically nonsignificant differences at* P*≥.05.

b The linear mixed-effects model was used to examine the effect of trial order on each AUC.

c iAUC: incremental area under the curve.

d nAUC: net area under the curve.

## Discussion

The present study demonstrated that inhibition of SAA by oral acarbose attenuated the early increase in post–starch-load BG at 30 min by approximately 10 mg/dl, which is clinically paramount, as this time point serves as an outstanding predictor of type 2 diabetes [6,7]. The BG peak was delayed by 30 min, leading to no significant difference in BG AUCs between T2 and T3.

Chewing acarbose with rice can introduce a delay in intestinal glucosidase inhibition compared to the immediate swallowing in T2. Such a delay would cause postprandial glucose excursion in T3 due to uninhibited early intestinal digestion [8]. However, our results paradoxically demonstrated that T3 significantly reduced 30-min postprandial blood glucose levels. This clear superiority of T3, despite the procedural delay, indicates that the immediate inhibition of salivary amylase in the oral cavity exerts a dominant, upstream suppressive effect on starch digestion, neutralizing any minor pharmacokinetic lag in the lower gastrointestinal tract.

The present findings may be consistent with an earlier study showing that swallowing food without chewing, which prevents oral SAA, reduces postprandial glycemia [9]. Although the method of acarbose administration used in this study is the same as the usual method of taking orally disintegrating tablets, this approach allowed direct examination of the contribution of oral starch digestion to postprandial glucose responses. Because blood insulin and incretins were not measured, it remains unclear how they reacted in T3, which should be investigated in a future study.

In conclusion, oral salivary amylase, which can be inhibited by acarbose, may influence the early phase of postprandial BG in healthy individuals.

## Supplementary material

10.2196/98441 Multimedia Appendix 1 Effect of oral acarbose on salivary α-amylase (SAA) activity in the pretest. SAA was substantially inhibited by 50 mg acarbose in all participants (before: 36.3, SD 21.8 U/L; after chewing acarbose in the mouth: 8.3, SD 0.9 U/L; paired *t* test, *P*=.0002). Each symbol from A to O represents 1 of the 15 participants.

10.2196/98441 Multimedia Appendix 2 Changes in salivary α-amylase after starch loading. Symbols and bars indicate means and SEs. No significant differences were observed between trials at any time point. The earliest peak from baseline occurred at 30 minutes in T3, at 60 minutes in T4, and at 120 minutes in T1 but was not observed in T2 (Student-Newman-Keuls test). In the linear mixed-effects model, the effect of trial on ΔSAA at 30 and 60 minutes was not statistically significant (*P*=.77 and *P*=.81). The carryover effect of trial order was likewise nonsignificant (*P*=.94 and *P*=.60).

10.2196/98441 Checklist 1 CONSORT checklist.

## Abbreviations

AUC: area under the curve
BG: blood glucose
SAA: salivary α-amylase
T1: trial 1
